# Imaging assessment after pancreaticoduodenectomy: reconstruction techniques—normal findings and complications

**DOI:** 10.1186/s13244-022-01306-4

**Published:** 2022-10-20

**Authors:** Lautaro Manuel Florentin, Gonzalo Dulcich, Roy López Grove, José Ignacio Paladini, Juan Carlos Spina

**Affiliations:** 1 Department of Radiology, Hospital Italiano de Buenos Aires, Buenos Aires, Argentina; 2 Department of General Surgery, Hospital Italiano de Buenos Aires, Buenos Aires, Argentina

**Keywords:** Pancreaticoduodenectomy, Pancreatic fistula, Pancreatic cancer, Pancreaticojejunostomy, Hepaticojejunostomy

## Abstract

Pancreaticoduodenectomy represents a major surgery for tumors located at the pancreatic head and the ampullary/periampullary region. This complex procedure is associated with a high morbidity rate. Many surgical techniques have been proposed in order to reduce mortality rates, although post-procedure complications represent a current problem. Different imaging findings and complications may appear depending on the surgical technique used. Hence, radiologists should be familiarized with them to distinguish normal findings from real complications. The most challenging scenarios are represented by abdominal fluid collections, and tumor recurrence, that may frequently mimic normal postsurgical changes.

## Key points

- MDCT represents the first-line imaging modality after pancreaticoduodenectomy.
- Most challenging complication diagnoses are pancreatic fistula and tumor recurrency.
- Additional angiographic-CT phase is recommended when vascular complications are suspected.
- MRI with gadoxetic-acid is recommended to detect hepaticojejunostomy stenosis and biliary fistula.
- DWI helps differentiate local recurrence form postsurgical changes when CT is inconclusive.

## Background

Pancreaticoduodenectomy is a major surgery for tumors located at the pancreatic head and the ampullary/periampullary region, and represents the only curative procedure available to date for this group of diseases. This complex procedure has been associated with a high morbidity rate [1]. Due to surgical techniques improvement, mortality rates have been significantly reduced, although post-procedure complications represent a frequent problem [2]. As there are different surgical techniques, each one with specific post-surgical imaging findings, the radiologist must be familiar with them in order to make a correct diagnosis and guide the patient's follow-up.

The aim of this article is to review the main pancreatic, gastric and bilioenteric reconstruction techniques used after a pancreaticoduodenectomy, illustrating the normal imaging findings and their complications.

## Surgical technique

**Fig. 1:**
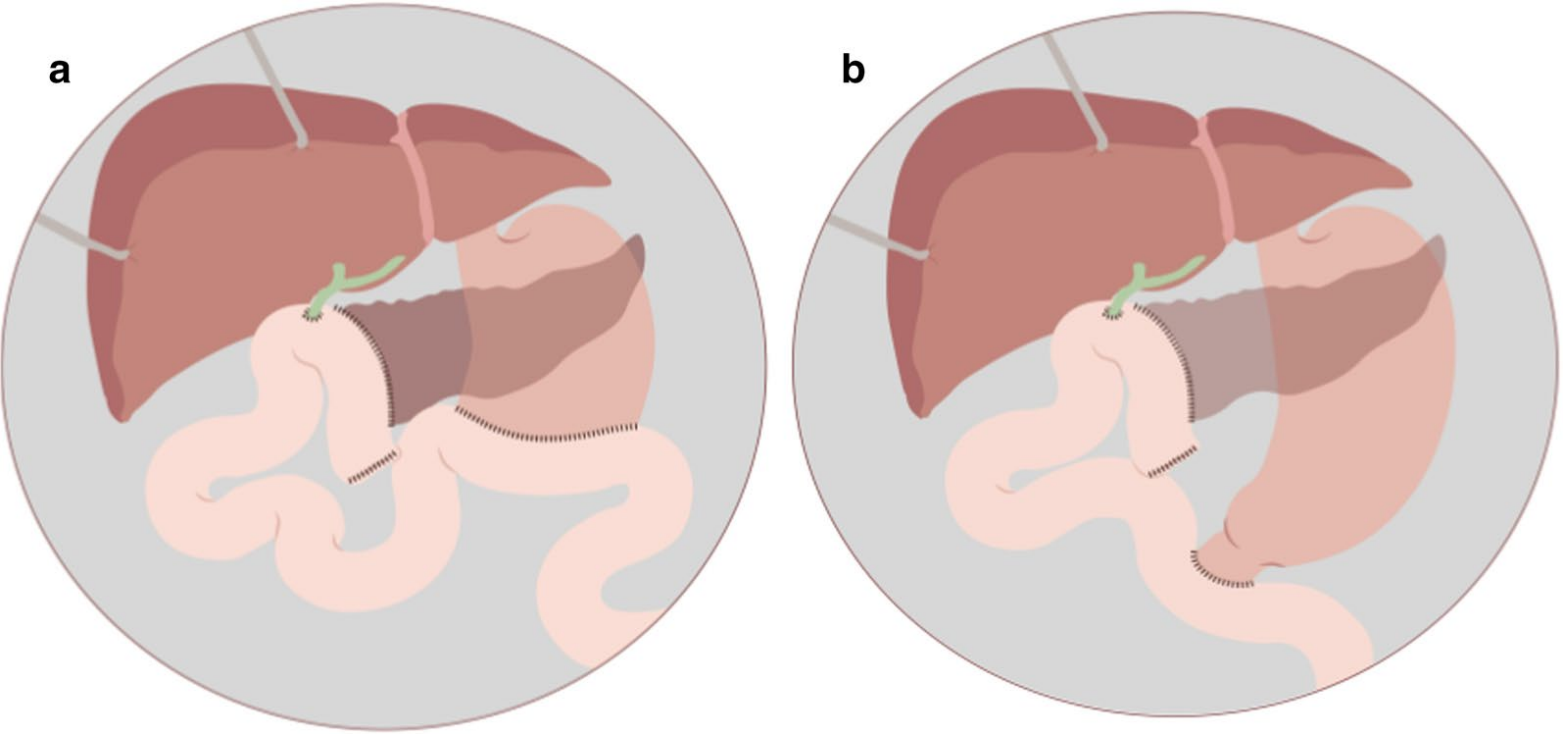
Pancreaticoduodenectomy: (**a**) Classic procedure (Whipple’s) (**b**) Pylorus-preserving procedure

Pancreaticoduodenectomy consists of two phases: the resection phase that involves the resection of pancreatic head, duodenum, gallbladder, distal bile duct and proximal jejunum, with or without pylorus preservation, followed by the reconstruction phase with the creation of three anastomosis: pancreaticojejunostomy, hepaticojejunostomy, and gastrojejunostomy (Whipple procedure) or duodenojejunostomy [3] (Fig. 1).

Computed tomography (CT) represents the main imaging modality in postsurgical assessment of pancreaticoduodenectomy due to its capability to explore the whole abdominal cavity with a high spatial resolution and short acquisition time. For routine post-surgical follow-up, we suggest a non-contrast phase to depict high density findings (surgical clips, pancreatic stent, and blood) and a portal venous phase (60–70 s delay) including the pelvis for a global assessment. Positive oral contrast agent is routinely used for postsurgical intestinal anatomy distinction, specially the efferent limb and gastroenteroanastomosis. Protocol should be based on clinical findings during a complicated postoperative period, with an abdomino-pelvic non-contrast phase for hemorrhagic fluid detection and an additional CT-angiographic phase if vascular complication is suspected. In cases of abdominal collection, a late phase after three minutes may improve its distinction. The use of neutral oral agents, such as water, is not recommended as bowel loops may mimic a collection.

In order to improve imaging surveillance quality, magnetic resonance imaging (MRI) should be considered an additional method to CT for several reasons. Firstly, it is well known that MR provides a higher pancreatic and hepaticojejunostomy evaluation, especially when MR cholangiopancreatography (MRCP) or hepatobiliary contrast agents are used [4]. Secondly, MR can better distinguish tumor recurrence from other normal conditions such as collapsed loop, since it better depicts endoluminal content, or perivascular cuffing and other mass-like imaging in the surgical bed based on diffusion weighted imaging (DWI).

### Pancreatic–enteric anastomosis

Pancreatic–enteric anastomosis represents the most challenging anastomosis during the surgical procedure and leads to most post-procedure complications [5]. There are two main pancreatic–enteric anastomosis techniques: pancreaticojejunostomy (PJ) and pancreatic-gastro (PG) anastomosis.

**Fig. 2:**
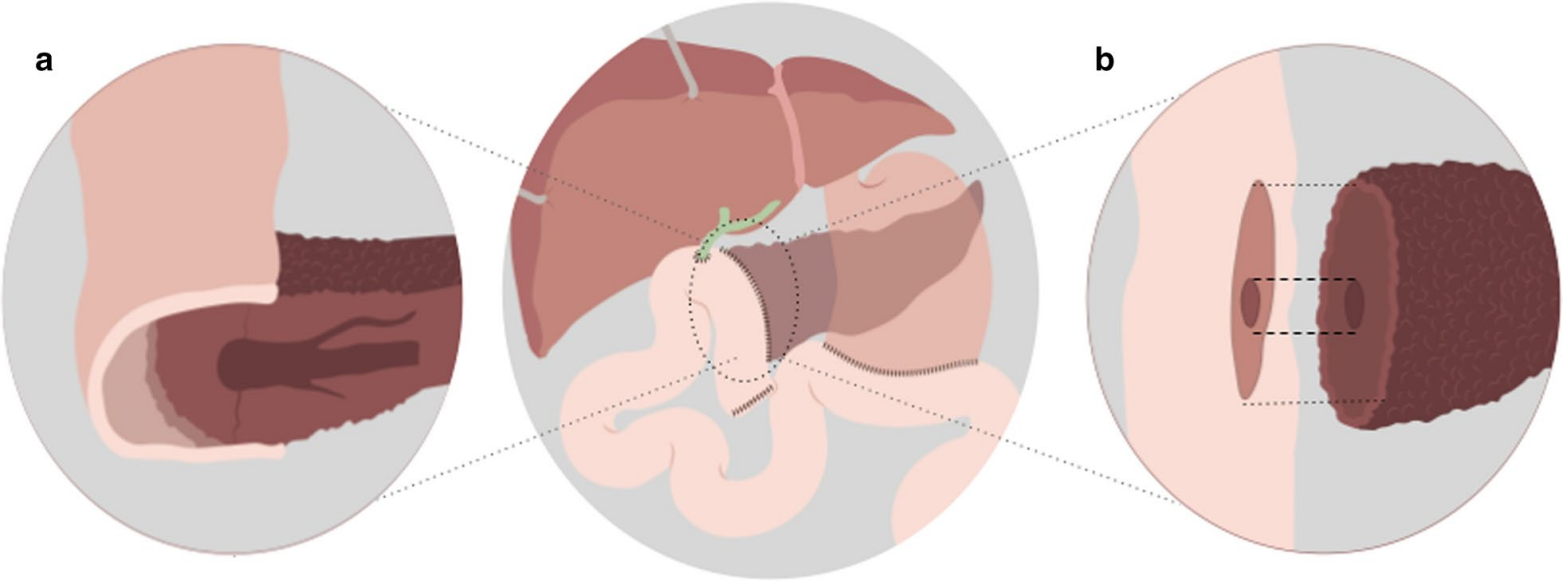
Pancreaticojejunostomy. Main surgical techniques: (**a**) Invagination (**b**) Duct-to-mucosa

**Fig. 3:**
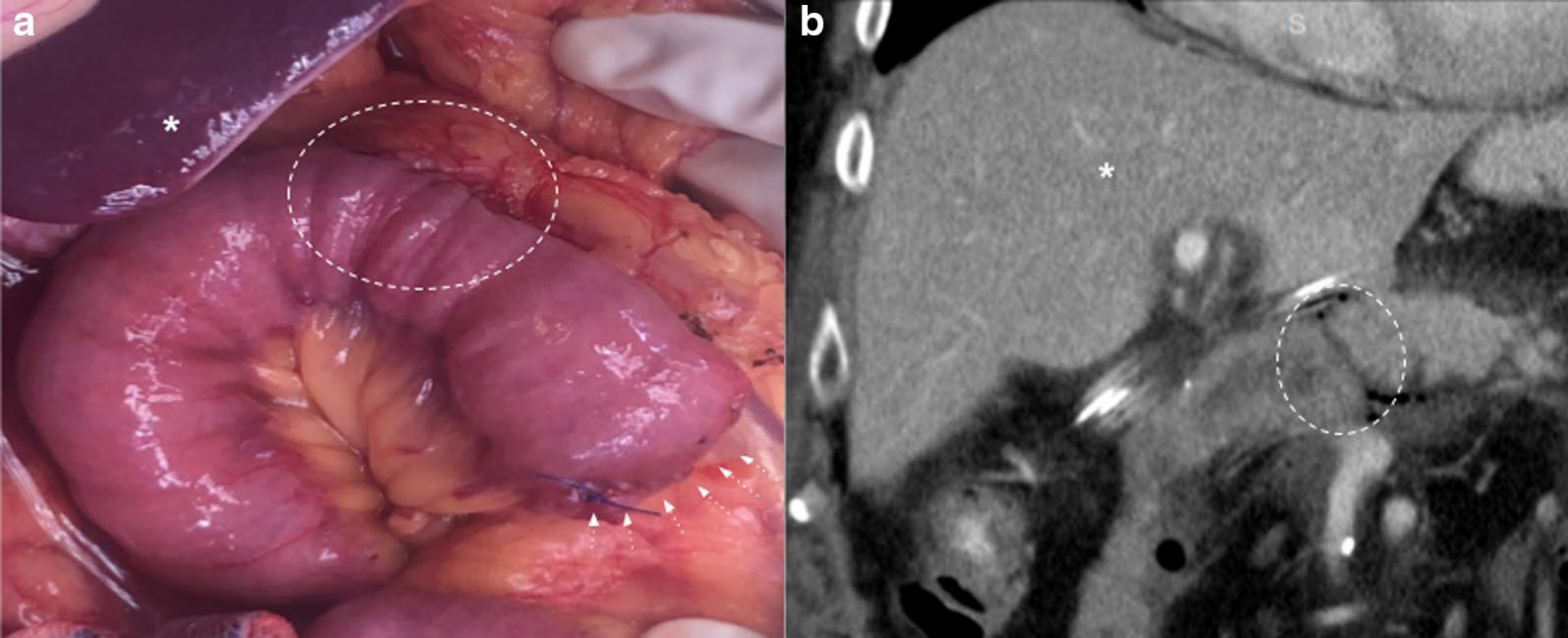
Pancreaticojejunostomy. Duct-to-mucosa. CT-surgical correlation: (**a**) Surgical photograph. (**b**) Contrasted Coronal CT scan. References: Duct-to-mucosa pancreaticojejunostomy (discontinuous circle); blind segment of afferent limb (small arrows); liver (asterisk)

Different metaanalyses show there is no significant difference between PJ and PG anastomosis in terms of pancreatic fistula, mortality or morbidity [6, 7]. Although some authors report a slight reduction in pancreatic fistula development with PG [8]. In our institution, PJ is the most used anastomosis. Different surgical techniques are employed to create the PJ anastomosis; the most important are: duct-to-mucosa and invagination anastomosis, being the former the most frequently used (Figs. 2, 3).

### Hepaticojejunostomy

**Fig. 4:**
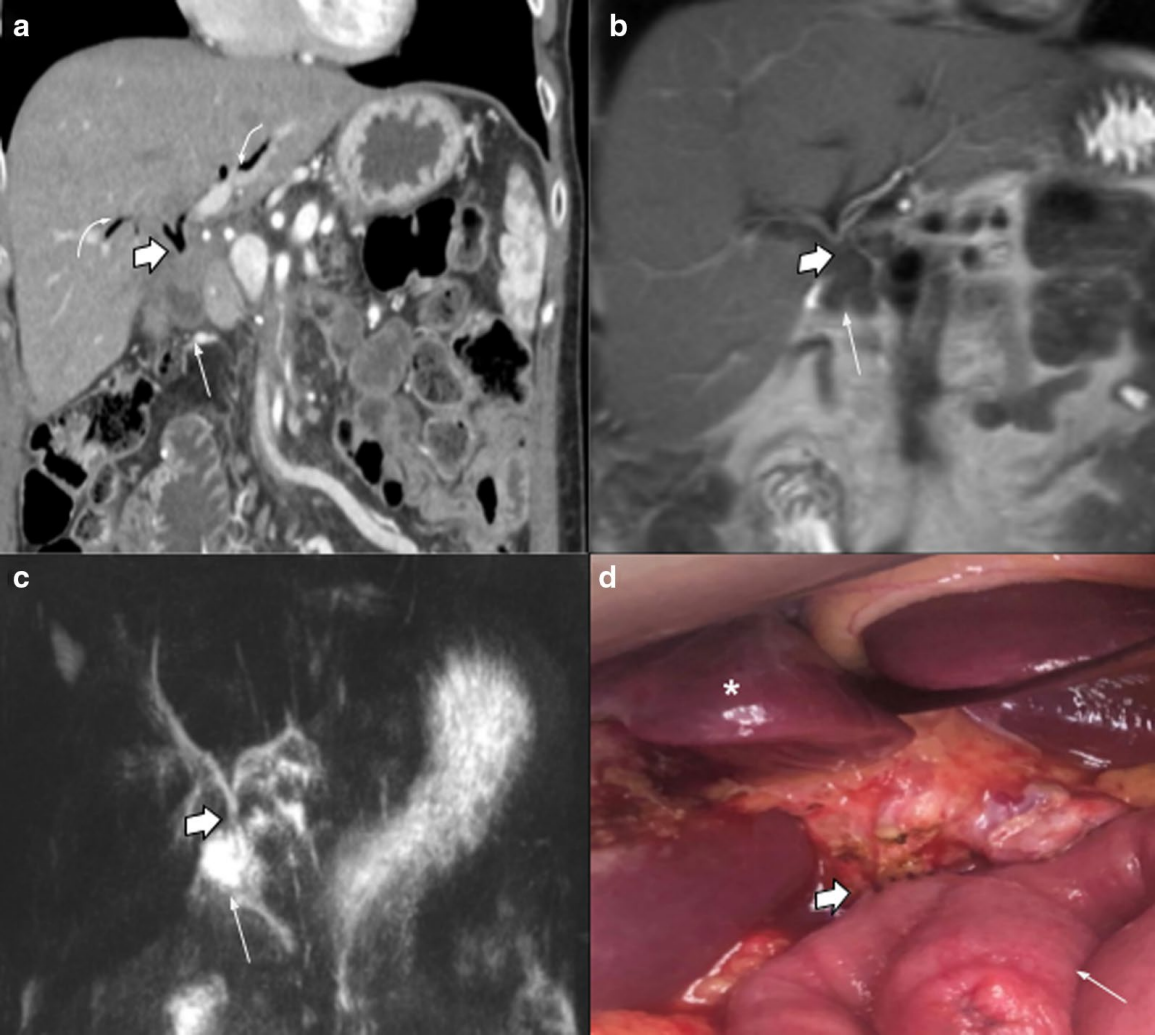
Hepaticojejunostomy. Radiologic-Surgical correlation: (**a**) Contrasted Coronal CT scan. (**b**) Coronal T2WI MRI image. (**c**) MRI Cholangiography. (**d**) Surgical photograph References: Partially collapsed afferent limb (white straight arrow). Pneumobilia (white curved arrow). Hepaticojejunostomy (thick arrow). Liver (asterisk)

Hepaticojejunostomy is usually located 20–30 cm distal to PJ. It may be difficult to find due to jejunal limb collapse. Pneumobilia is a normal postoperative finding that helps to identify it, as well as using the hepatic hilum as a landmark (looking for surgical clips). When positive oral contrast agent is used, sometimes reflux to the afferent limb may occur, so it can fill the anastomosis and helps to recognize it. The use of coronal plane reconstructions is recommended for a better visualization. MRCP and MRI with gadoxetic acid are the most useful methods to assess permeability (Fig. 4).

### Gastrojejunostomy

**Fig. 5:**
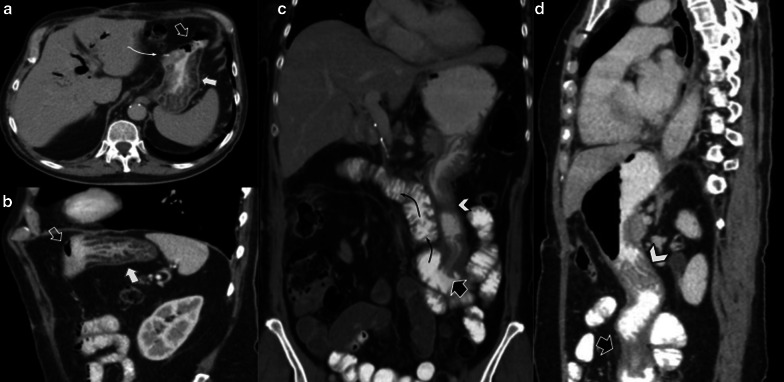
Gastrojejunostomy and duodenojejunostomy: (**a**, **b**) Gastrojejunostomy. Axial and sagittal portal venous phase CT scan, respectively. (**c**, **d**) Duodenojejunostomy. Coronal and oblique sagittal view portal venous phase CT scan, respectively. Note the importance of using positive oral contrast to denote marked mucosal pattern change in A and B, and to depict the pylorus and latero-terminal anastomosis in duodenojejunostomy. References: White curved arrow: Surgical clips; Thick white arrows: gastric remnant; black arrows: ascended jejunal loop; Thick black arrow: latero-terminal anastomosis; arrowhead: pylorus; curved black arrows: oral contrast reflux to afferent limb. Black arrows: ascended jejunal loop

Gastrojejunostomy allows reconstitution of the digestive tract. Depending on the primary pathology and the surgeon's choice, it can be performed with resection of the gastric antrum (classic Whipple procedure) or with pylorus preservation (pylorus preservation procedure). This anastomosis is usually located in the left upper quadrant. The use of a positive oral contrast agent is essential to digestive tract reconstruction assessment, allowing delineation of marked change in mucosal pattern in gastroenteric anastomosis and pylorus identification, when preserved (Fig. 5).

### Intestinal reconstruction

**Fig. 6:**
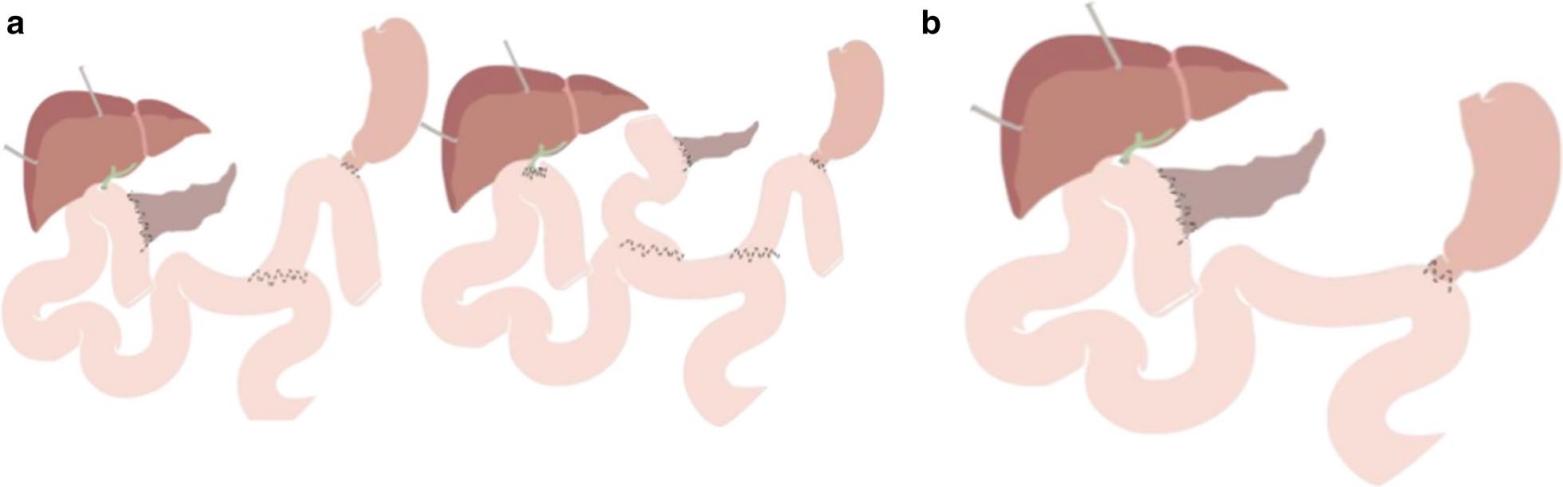
Intestinal reconstruction techniques: (**a**) Y de Roux intestinal reconstruction. Left: classic type; right: Machado type. (**b**) Child type reconstruction

Classic procedure includes intestinal reconstruction by an unique jejunal loop where the three anastomoses are performed. This technique is known as *Child type* reconstruction. Another popular intestinal reconstruction technique is the *Roux-en-Y* type, in which a jejunal loop is constructed in order to make the pancreaticojejunal and biliodigestive anastomosis. This jejunal loop is called afferent or biliodigestive limb. In our institution, a Roux variant known as *Machado type* is used, which allows to reduce morbidity and mortality in case of pancreatic or biliary fistula, preserving each anastomosis separately. Intestinal reconstruction techniques are exposed in Fig. 6.

## Normal postoperative findings and complications

In this section, normal postoperative findings of each anastomosis or intestinal reconstruction technique, as well as their complications will be discussed (Table 1).

**Table 1:** Normal findings and complications

Normal findings	Complications		
	Postoperative Anatomy	Surgical techniques	Related Complications
Fluid, edema and fat stranding	Pancreaticojejunostomy	Duct-to-mucosa Invagination Use of tutor	Pancreatic Fistula Stricture Tutor migration
Perivascular cuffing	Biliary-enteric Anastomosis	Hepaticojejunostomy Chledochojejunostomy	Biliary fistula (Bilom) Stricture
Lymph nodes	Efferent limb (Alimentary limb)	Gastrojejunostomy Duodenojejunostomy	Delayed gastric emptying Stricture
Pancreatic duct dilatation	Afferent limb (Biliary or Roux limb)	Child Y de Rox	Afferent limb syndrome
Mild intrahepatic biliary dilatation	Vascular	–	Hemorrhage Pseudoaneurysm Hepatic Infarct Venous thrombosis
*Tumor recurrence*			

### Normal postoperative findings

**Fig. 7:**
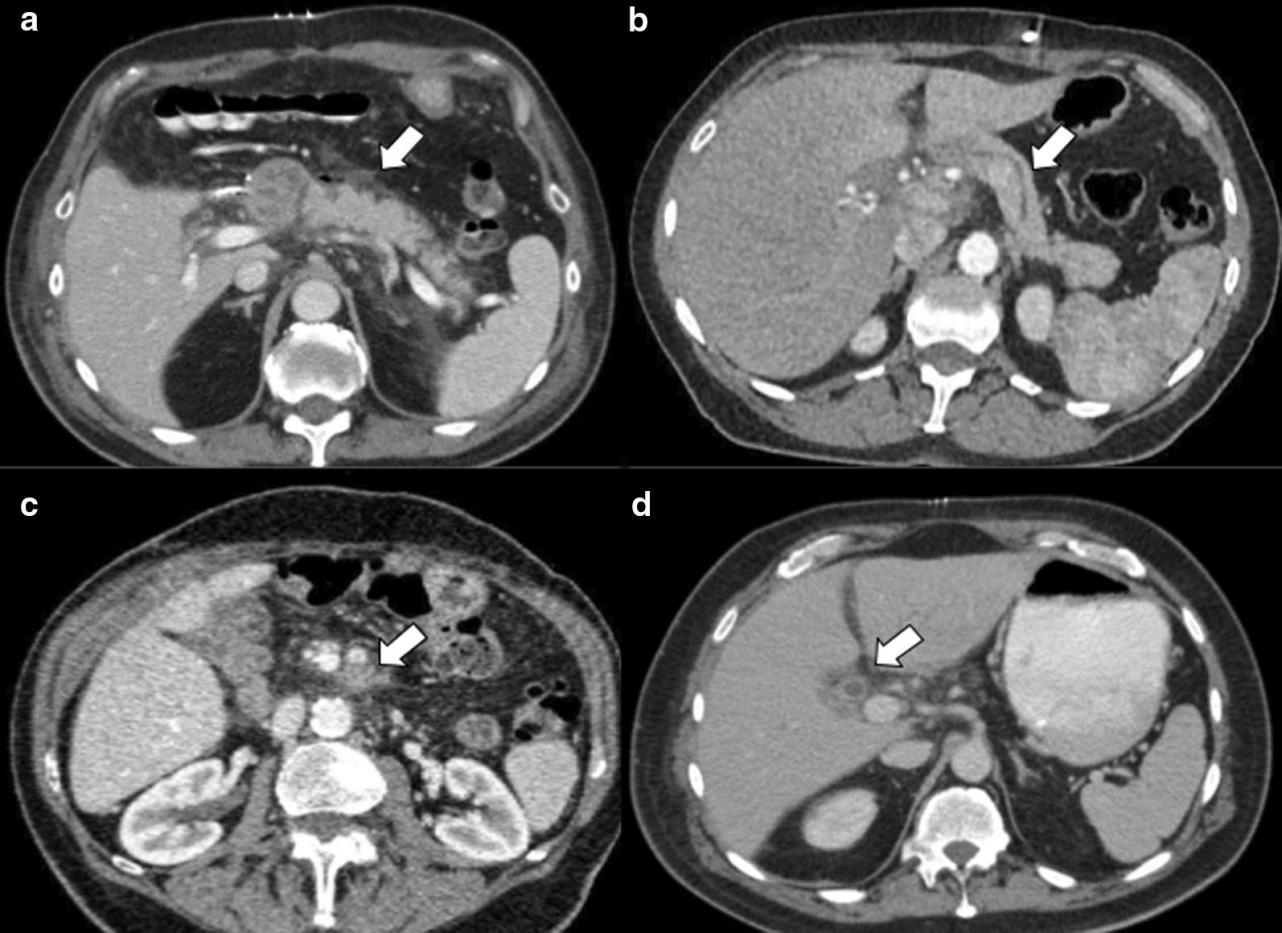
Normal postoperative findings: (**a**) Peripancreatic fat stranding; (**b**) Mild pancreatic duct dilatation; (**c**) Perivascular cuffing; (**d**) Intrahepatic biliary duct enhancement and dilatation

During the early perioperative period, imaging may show temporary findings related to surgery that should not be misunderstood as tumor recurrence or abnormal inflammatory processes. These findings include fluid, edema and peripancreatic fat stranding, pneumoperitoneum, pneumobilia and pneumowirsung, mild pancreatic duct and/or intrahepatic biliary dilatation, lymph nodes and perivascular cuffing (Fig. 7).

#### Tumor recurrence mimics: imaging challenge

Normal postoperative findings may mimic tumor recurrence. Lymphadenopathy, collapsed loop, perivascular cuffing and pancreatic and/or intrahepatic ducts dilatation represent the major challenges.

##### Lymphadenopathy

Lymphadenopathy represents one of the most frequent postoperative findings. Adenopathies are expected to regress within the first six months after surgery, but can sometimes persist for longer periods [9].

**Fig. 8:**
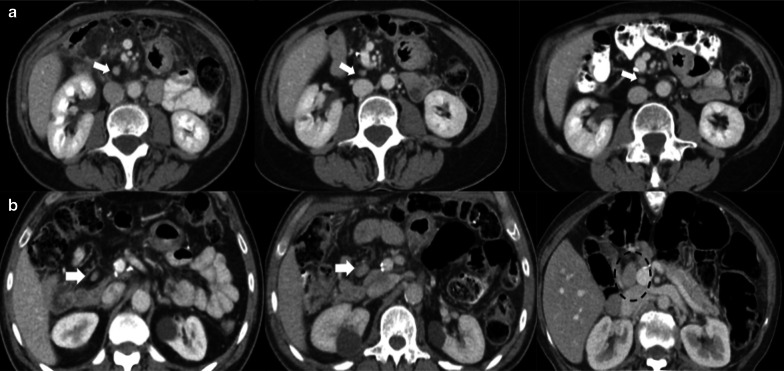
Lymphadenopathy vs. Tumor recurrence. CT challenge. Two different patients (**a**) 63-year-old female patient with persisting lymph node along 2-year follow-up. Six months, one- and two-years CT scans, respectively. Note the decreasing size of the node over time (big arrow). Clinical and biological markers level stable. (**b**) 65-year-old male patient along annual CT scan controls. An increasing lymph node is seen during the follow-up (large arrow). Note the rounded shape appearance and the short axis size greater than 10 mm (dotted circle) after three years from surgery. Tumor recurrence was proven by biopsy in patient B

Although morphological characteristics such as short axis less than 1cm and oval shape can help identify reactive lymph nodes in the early period, the new-formed or growing nodes should raise suspicion for malignancy [10] (Fig. 8).

##### Collapsed loop

**Fig. 9:**
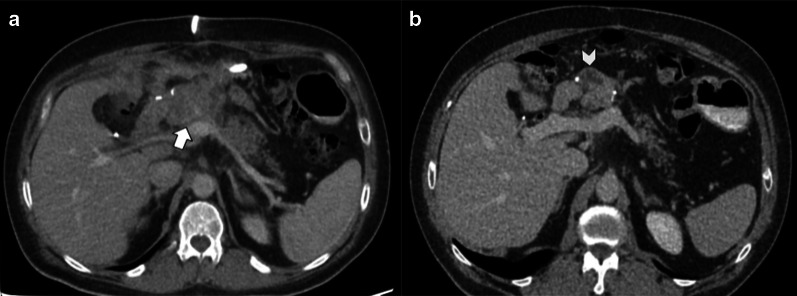
Collapsed loop versus Tumor recurrence. CT challenge. 58-year-old male patient with history of pancreaticoduodenectomy due to periampullary tumor. Control CT scans. (**a**) Heterogeneous, ill-defined image near pancreaticojejunostomy (big arrow) with peripheral fat stranding and contrast enhancement is seen after three months from surgery. No clinical symptoms or biological marker elevation were present. (**b**) 18 months CT scan control revealed total fat stranding resolution and demonstrated that the ill-defined enhancing image was due to a collapsed loop, which appears mild distended with normal fluid contain (arrowhead)

**Fig. 10:**
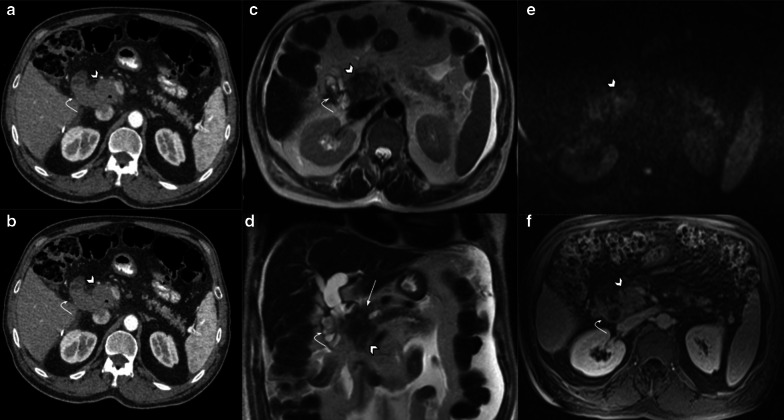
Collapsed loop vs tumor recurrence. The MRI role. 58-year-old male patient during routine follow-up after pancreaticoduodenectomy due to pancreatic adenocarcinoma. (**a**, **b**) Control CT scan. Well-defined enhancing soft tissue image (arrowhead) between the afferent loop (curved arrow) and the pancreaticojejunostomy. Collapsed loop versus tumor recurrence were suggested as possible diagnosis, so MRI was indicated. (**c**, **d**, **e**, **f**) MRI performed three days later revealed a heterogeneous ill-defined mass located between afferent loop (curved arrow) and pancreaticojejunostomy (straight arrow in** d**). Note the better tissue differentiation of MRI over CT, ruling out collapsed loop diagnosis. Restriction on DWI (**e**) and contrast enhancement (**f**) was also noted (arrowhead in** e** and** f**). Local recurrence was confirmed by PET-CT

**Fig. 11:**
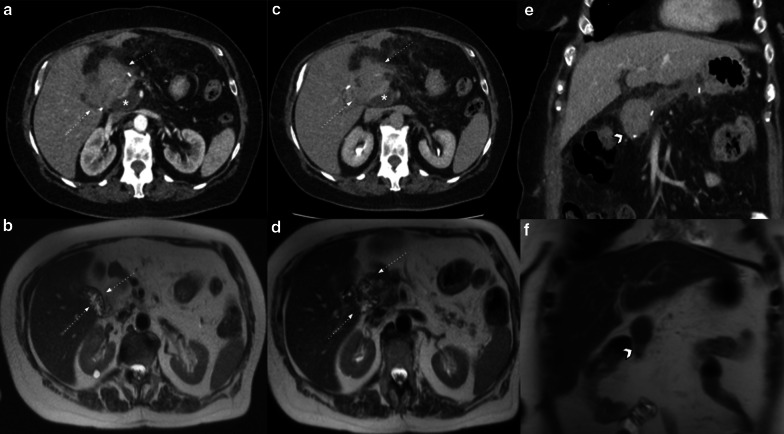
Collapsed loop vs tumor recurrence. The MRI role. 62-year-old female patient during routine follow-up after pancreaticoduodenectomy due to pancreatic adenocarcinoma three months early. (**a**, **c**, **e**) Axial and coronal view contrast-enhanced CT scan. Homogeneous fluid collection (asterisk) in the surgical bed and related to an ill-defined soft tissue enhancing image with peripheral fat stranding associated (dotted arrows). Coronal view denotes a pseudo-mass image (arrowhead in** c**). Collapsed loop versus inflammatory changes were suggested as possible diagnosis, so strict control was indicated. (**b**, **d**, **f**) MRI performed two months later. Prior collection complete resolution. The ill-defined image related to the hepatic hilum is now better defined as the collapsed loop due to their fluid content and intestinal folds (dotted arrow). Coronal view denotes the blind portion of the afferent loop as the previously described pseudo-mass image (arrowhead in** f**)

Collapsed loops may be difficult to evaluate in CT scans. The *mass-like* appearance makes it difficult to rule out recurrence (Fig. 9). Multiplanar reconstructions can help by following the loop beyond the surgical bed in many cases. In doubtful cases, MRI can be chosen as an imaging follow-up method to differentiate from tumor recurrence as it can usually determine the fluid content of the loop, thus differentiating it from tumor recurrence (Figs. 10 and 11). For this purpose, we recommend the addition of thin axial and coronal T2 weighted images (3 mm) in order to better depict intestinal folds which are clearly visible in the yeyunal wall of a collapsed loop and absent in case of a recurrence.

##### Perivascular cuffing

**Fig.12:**
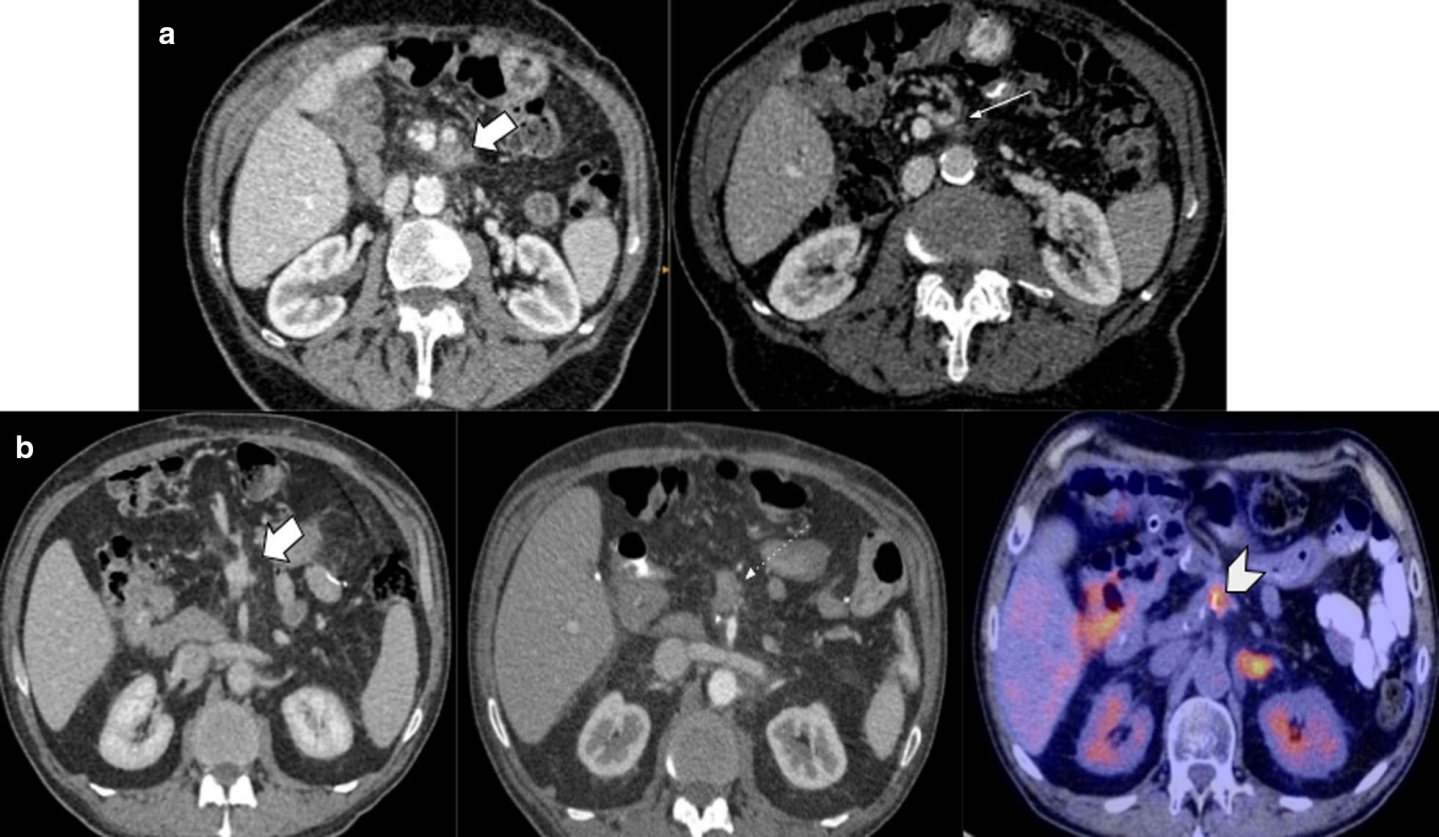
Perivascular cuffing vs. tumor recurrence. CT challenge. Two different patients (**a**) 81-year-old male patient three weeks after pancreaticoduodenectomy due to ampulloma. (**b**) 45-year-old male patient with fever 21 days after pancreaticoduodenectomy due to adenocarcinoma. Soft-tissue imaging in the surgical bed (thick white arrows), in both cases strict follow-up was required. In patient A, the focal fat stranding has markedly resolved (thin arrow): while in patient B persists without significant change after a year (dotted arrow). PET/CT shows hypercaptation mass, which confirms tumor recurrence (arrowhead)

**Fig. 13:**
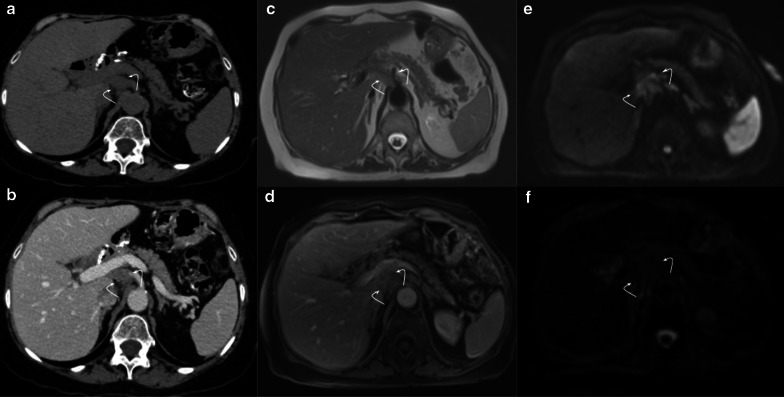
Tumor recurrence. DWI value. 69-year-old female patient during routine follow-up after pancreaticoduodenectomy due to pancreatic adenocarcinoma. (**a**, **b**) Non-contrast and portal venous phase CT scan, respectively. Shows an ill-defined soft tissue enhancing image in the surgical bed (curved arrows). (**c**, **d**, **e**, **f**) Six month later MRI control. Persistent image surrounding the celiac trunk with no significant progression (curved arrows in** c** and** d**). DWI (**e**) and ADC map (**f**) shows restriction, raising local recurrence diagnosis (curved arrows in** e** and** f**). The patient evolved unfavorably with disease progression

Perivascular cuffing is characterized by the presence of soft-tissue stranding in mesenteric fat that occurs within the surgical bed and surrounding celiac axis; it is due to a transient inflammatory reaction. It may appear as a mass-like image and can be easily mistaken for residual disease on CT scan (Fig. 12). MRI, especially with the use of DWI, can help differentiate this transient and benign finding from tumor recurrence when CT is inconclusive (Fig.13).

##### Pancreatic and/or intrahepatic ducts dilatation

**Fig.14:**
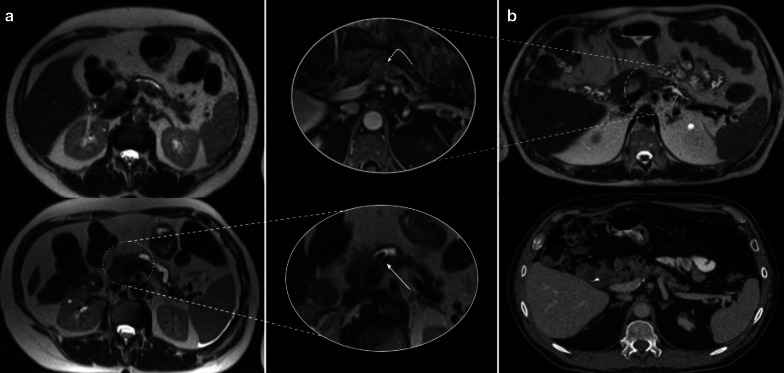
Benign duct dilatation vs. tumor recurrence. (**a**) 60-year-old female patient during acute abdominal pain. MRI showed pancreatic duct dilatation with filling defect (white straight arrow) just proximal to pancreaticojejunostomy. Acute stump pancreatitis was the final diagnosis. Endoscopic ultrasound confirmed mucoid plug related to pancreaticojejunostomy. Previous MRI (upper) denoted normal wirsung caliber. (**b**) MRI scan control in a 68-year-old male patient with pancreaticoduodenectomy due to pancreatic cancer. MRI performed three years after surgery reveals increased pancreatic duct dilatation related to an ill-defined hypovascular focal image in the pancreas body (curved arrow in magnified image). Lower: prior CT scan control denotes normal wirsung caliber. Endoscopy ultrasound guided biopsy confirmed adenocarcinoma recurrence

Mild biliary and pancreatic dilatation could be a persistent normal finding. Significant dilatation, or progression of the known one, should raise suspicion of local recurrence near the pancreaticojejunostomy or anastomotic stricture (Fig.14).

As exposed above, tumor recurrence is a diagnosis challenge. At this point, the use of MRI with DWI improves the differentiation between early local recurrence from post-surgical changes: the presence of mass-like tissue on surgical bed with restriction at DWI with low ADC map values is suggestive of tumor recurrence [11]. MRI has proven to be a highly sensitive method in oncological follow-up, and the correct interpretation of DWI is key to detecting small lesions that are not visible with other imaging methods [12, 13].

At least, it is important to highlight that strict follow-up and combination with serum CA 19-9 is essential to confirm recurrence. If doubt exists after using the imaging modalities we have discussed, 18 FDG PET-CT can help to confirm or dismiss the diagnosis.

Tumor recurrence is the main cause of poor long-term outcome after pancreaticoduodenectomy, so radiologist’s awareness must be elevated: do not forget to use as many prior scans as available, especially the earliest postoperative scan.

### Complications

#### Pancreaticojejunostomy

It is important to recognize the anastomosis type (PG vs PJ), because it is the main site of complications. The most frequent one is pancreatic fistula (PF), with a global incidence of 4 to 30%. It is associated with increased length of hospital stay and mortality [4].

**Fig. 15:**
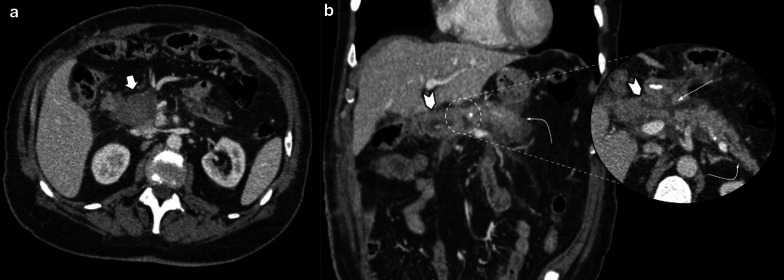
Pancreatic fistula, (**a**, **b**) Axial and coronal portal venous phase CT scan, respectively. 72-year-old male patient with pancreaticoduodenectomy due to neuroendocrine tumor, during second-month control CT scan. Homogeneous collection (big arrow) in surgical bed. That collection impresses to be in contiguity with the PJ (straight arrow). Normal pancreatic stump (curved arrow) and afferent loop (arrowhead) are also noted

PF is a clinical diagnosis characterized by the presence of amylase concentration three times higher in fluid collection than in serum, and/or persistence of it 7–10 days after surgery. At CT, it appears as a fluid collection near the PJ (Fig. 15).

**Fig. 16:**
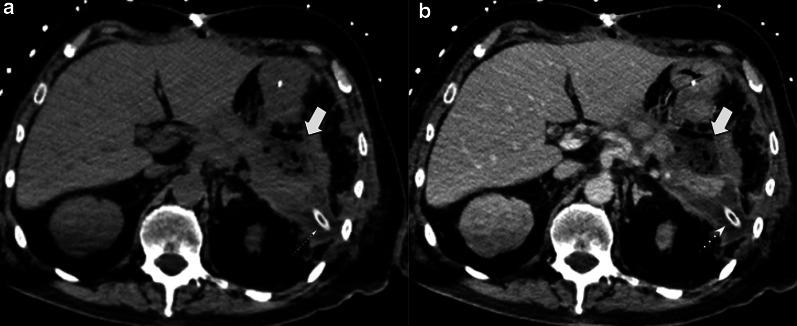
Abdominal abscess (**a**) Axial non-contrast CT scan. (**b**) Axial portal venous phase CT scan. 52-year-old female patient with fever after 20 days of pancreaticoduodenectomy due to mucinous cystic tumor. CT scan showed heterogeneous fluid collection with air content (big arrow). Drainage (dotted arrow) analysis disclosed purulent material

**Fig. 17:**
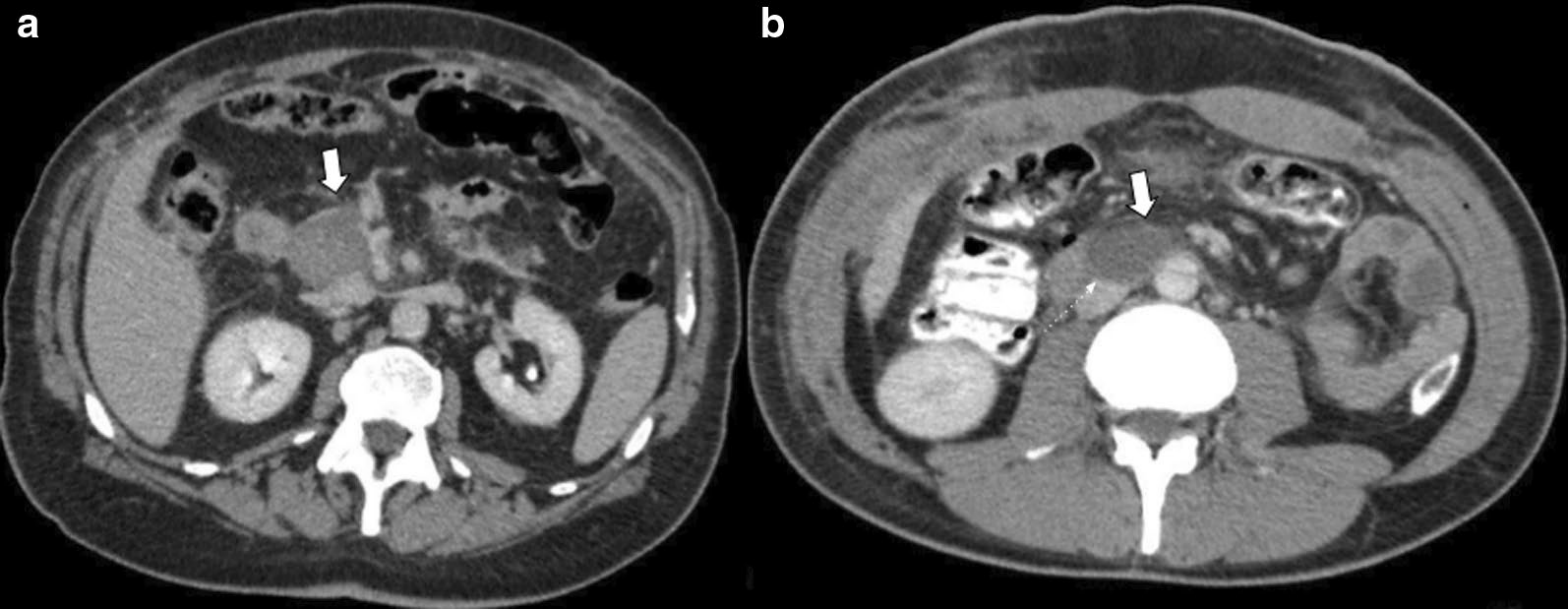
Pancreatic fistula vs. Abscess. Two different patients. Axial portal venous phase CT scans: (**a**) 73-year-old male patient with abdominal pain 25 days after pancreaticoduodenectomy due to pancreatic neuroendocrine tumor. (**b**) 45-year-old male patient with fever 21 days after pancreaticoduodenectomy due to ampulloma. Both present an homogeneous fluid collection near the PJ (big arrow). In patient B impresses to exist a thin wall during portal phase (dotted arrow). Both of them were percutaneous drainage with elevated amylase concentration in A and purulent fluid in B.

When a fluid collection is seen near the PJ, it is important to identify whether it is an infected collection. PF usually is expected to resolve by itself, while an abdominal abscess requires drainage. CT features that suggest abscess include heterogeneity within the collection with or without air content, and the presence of an enhancing wall (Fig. 16). Nevertheless, radiologists should interpret these findings in conjunction with clinical history and laboratory data, because imaging appearance between PF and abscess usually overlap, representing a major challenge [4] (Fig. 17).

In order to reduce the risk of PF, a stent in the anastomosis may be placed, depending on the surgeon's criteria. Stent migration is a usual finding, with no pathological significance.

**Fig. 18:**
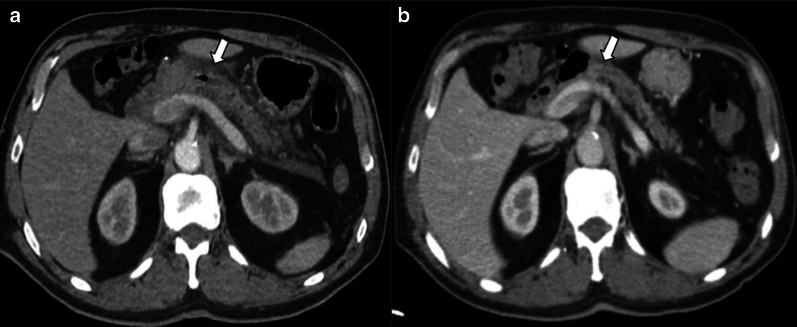
Pancreatojejunostomy stenosis and recurrent pancreatitis (**a**) 72-year-old male patient with history of pancreaticoduodenectomy due to chronic pancreatitis in the setting of acute stump pancreatitis (big arrow). Endoscopic ultrasound revealed pancreaticojejunostomy with decreased caliber. (**b**) Control CT scan performed three months later denote normal wirsung

Another complication to be aware of is anastomosis stenosis. PJ diameter may be reduced, either due to fibrosis or tumor recurrence. Stenosis predisposes to recurrent pancreatitis episodes, which lead to PJ assessment in order to achieve the etiology (Fig. 18).

#### Hepaticojejunostomy

**Fig. 19:**
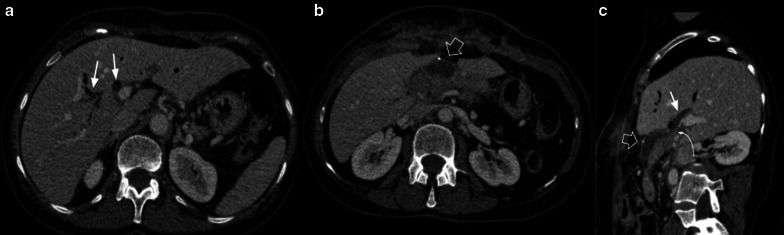
Biliary fistula 67-year-old female patient with increased fluid drainage after 60 days of pancreaticoduodenectomy due to ampuloma. (**a**, **b**, **c**) Axial and oblique sagittal portal venous phase CT scan. The intrahepatic biliary duct shows dilatation and fluid level within (white arrows in **a** and **c**). A collection in the surgical bed (empty arrow in** b** and** c**) and biliary connection (curved arrow in** c**) is also shown. Drainage analysis disclosed high bilirubin levels, which confirms the diagnosis of biliary fistula

Biliary fistula (BF) is a rare complication of hepaticojejunostomy, occurring in 3–4% of all procedures. The presence of a fluid collection adjacent to the anastomosis is a finding suggestive of BF in CT imaging [14] (Fig. 19).

**Fig. 20:**
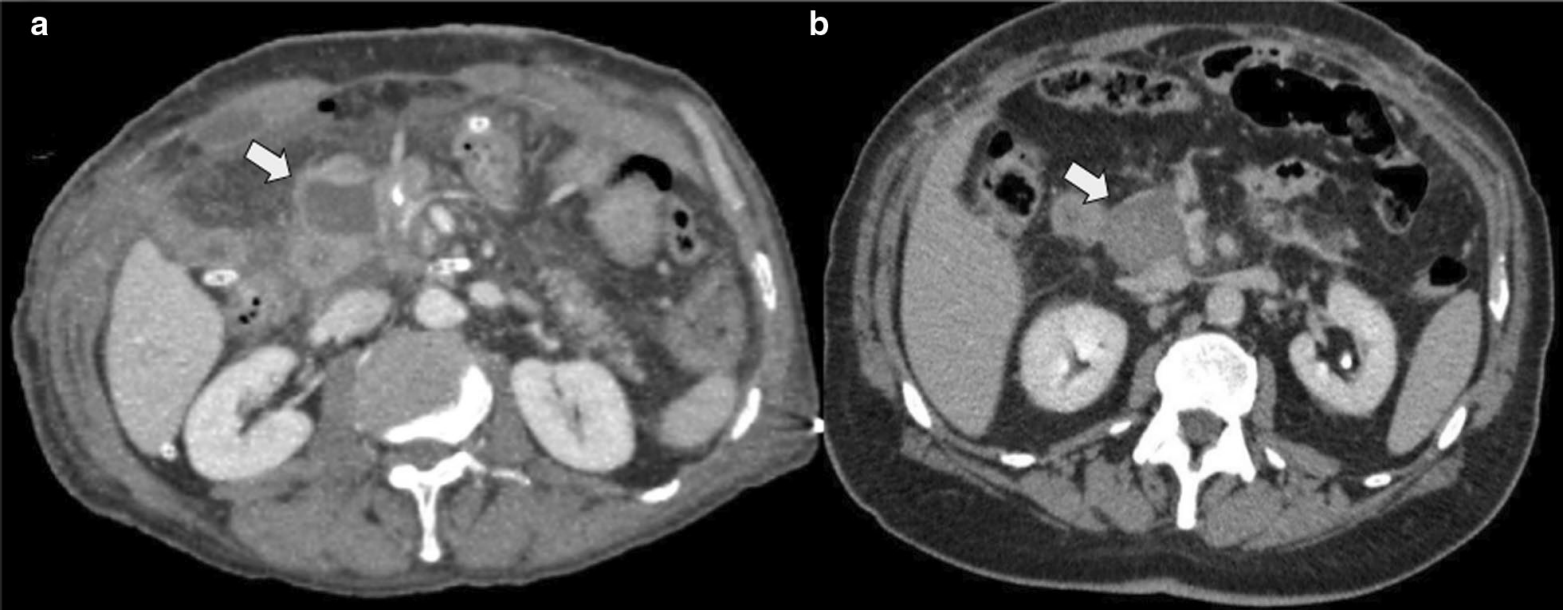
Biliary vs pancreatic fistula. Two different patients (**a**) 65-year-old male patient during abdominal pain three weeks after pancreaticoduodenectomy due to adenocarcinoma. (**b**) 50-year-old male patient with fever 21 days after pancreaticoduodenectomy due to ampulloma. Both patients presented fluid collection near the surgical bed, the both were homogeneous and no enhancement was present (big arrows). Fluid drainage analysis confirmed biliary fistula in** a** and pancreatic fistula in** b**. Note the similar imaging appearance, being indistinguishable one fistula from each other

As BF and PF depict similar imaging findings, and the proximity of the anastomosis (PJ and HJ) may not confirm the leak origin, biochemical analysis of fluid is essential for correct diagnosis: high bilirubin levels in fluid confirm BF [15] (Fig. 20).

**Fig. 21:**
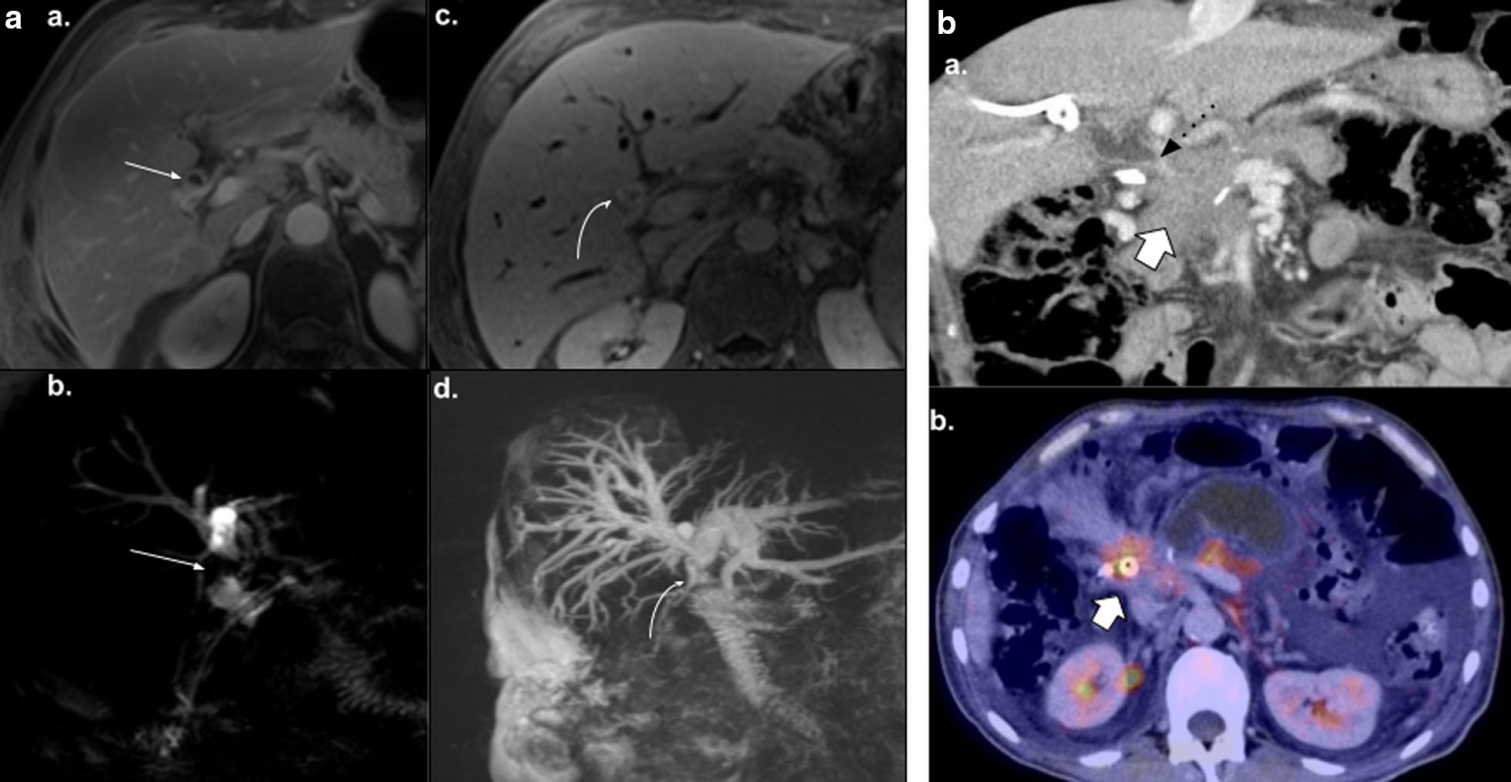
Hepaticojejunostomy stenosis. Two different patients (**a**) 67-year-old male patient with pancreaticoduodenectomy due to cholangiocarcinoma. All images below to normal postoperative control MRI. (a, b) one month after surgery (c, d) 7 months control. Note the caliber reduction in hepaticojejunostomy due to thickness of hepatic bile duct (curved arrows). Intraoperative cholangiography confirmed total hepaticojejunostomy stenosis (not shown) (**b**) 59-year-old male patient with abdominal discomfort and unexplained weight loss two years after pancreaticoduodenectomy owing to adenocarcinoma. CT scan denotes abrupt intrahepatic biliary stenosis (dotted arrow) due to ill-defined mass (big arrow). PET/CT fusion confirms local tumor recurrence as causal of stenosis

Stricture is another complication, commonly in late controls. Clinical presentation includes jaundice and cholangitis, in case it is not detected early [16]. As expected, imaging shows intrahepatic biliary tract dilatation. In those cases, tumor recurrence is the main diagnosis to be ruled out, due to treatment and prognosis implications [17] (Fig. 21).

**Fig. 22:**
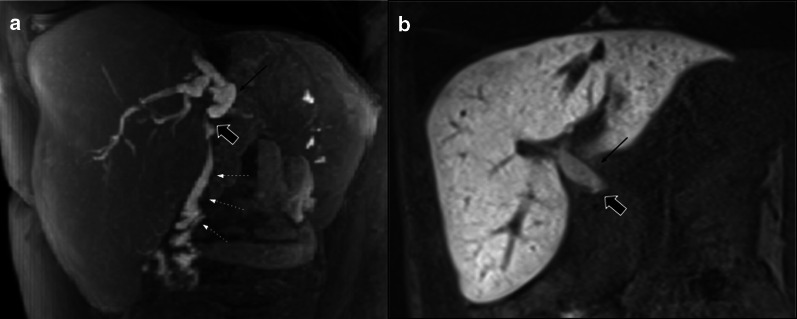
Hepatobiliary-specific contrast agent in hepaticojejunostomy assessment, Hepaticojejunostomy Permeability. Two different patients who underwent hepato-biliary surgeries with hepaticojejunostomy confection. MRI with hepatobiliary-specific contrast agent Gd-EOB-DTPA (gadoxetic acid) was performed due to postoperative biliary complication suspicion. (**a**, **b**) Hepaticojejunostomy (thick black arrows) permeability evaluation: MR Cholangiographic image shows afferent limb opacification (dotted arrows in** a**) that confirmed permeability, while stop in hepaticojejunostomy during hepatobiliary late phase (i.e., 5 h after IV agent injection) depict anastomosis stenosis. Common bile duct (thin black arrow)

The use of MRI with hepatobiliary-specific contrast agent (gadoxetic-acid) represents a safe and noninvasive option to detect postoperative biliary complications, such as hepaticojejunostomy stenosis and biliary fistula. Biliary excretion of contrast agent on hepatobiliary late phase (20 min) allows demonstration of anastomosis patency by identifying afferent loop filling or contrast leak into a neighboring fluid collection, allowing diagnosis of a biloma (Fig. 22). Sometimes, further delayed images (2 hours or more) must be acquired to achieve these diagnoses.

#### Gastrojejunostomy and intestinal reconstruction

As both gastrojejunostomy and intestinal reconstruction are intended to allow return of digestive tract transit after surgery, they are mentioned together.

**Fig. 23:**
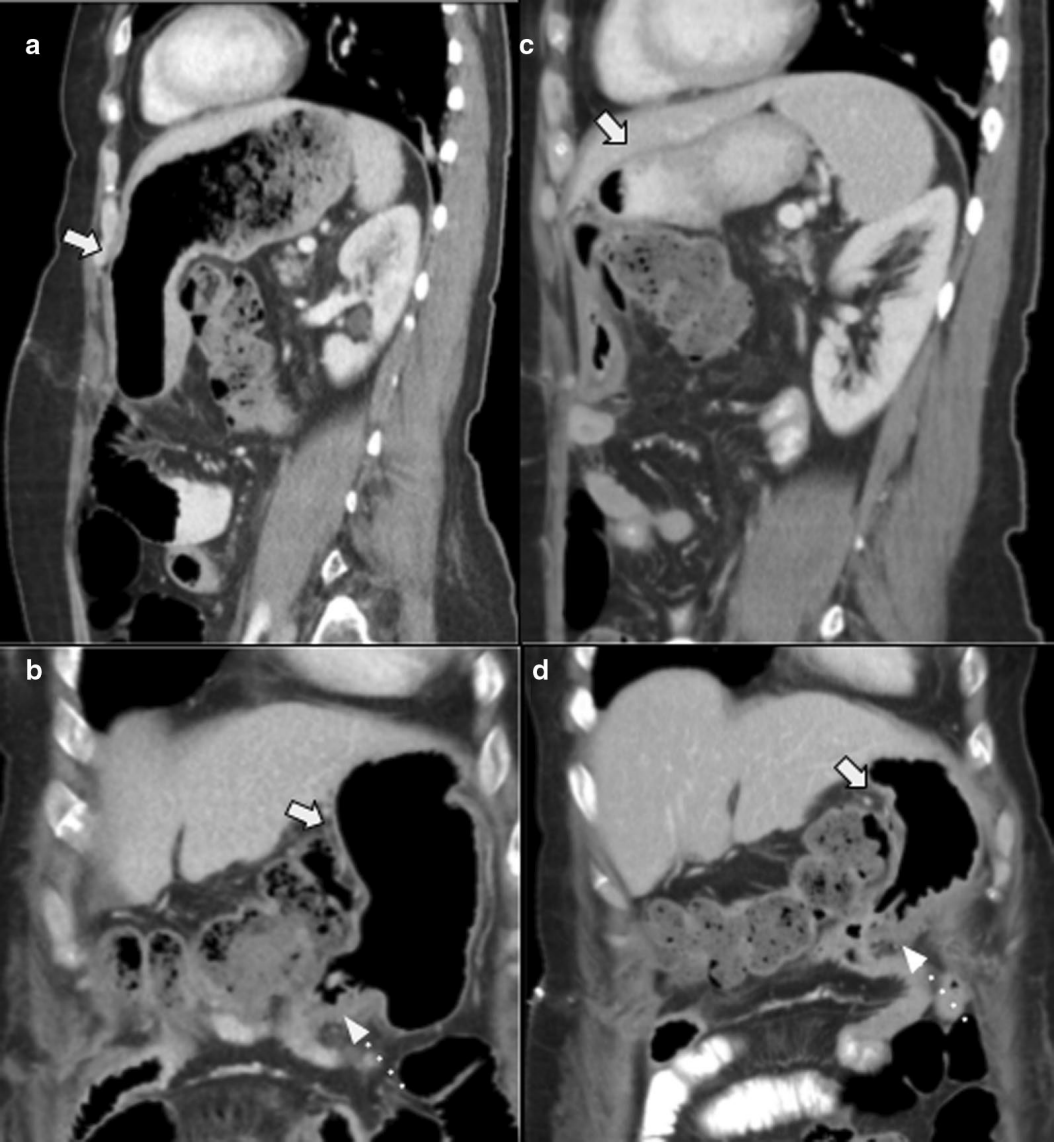
Delayed gastric emptying. 57-year-old female patient inability to feed orally with abdominal pain and distension 3 weeks after pylorus-preserving pancreaticoduodenectomy. (**a**, **b**) CT scans during acute event revealed marked stomach distention (big arrows). (**c**, **d**) Normal volume regression after medical treatment (big arrows). Duodenojejunostomy is also noted (dotted arrows in** b** and** d**)

**Fig. 24:**
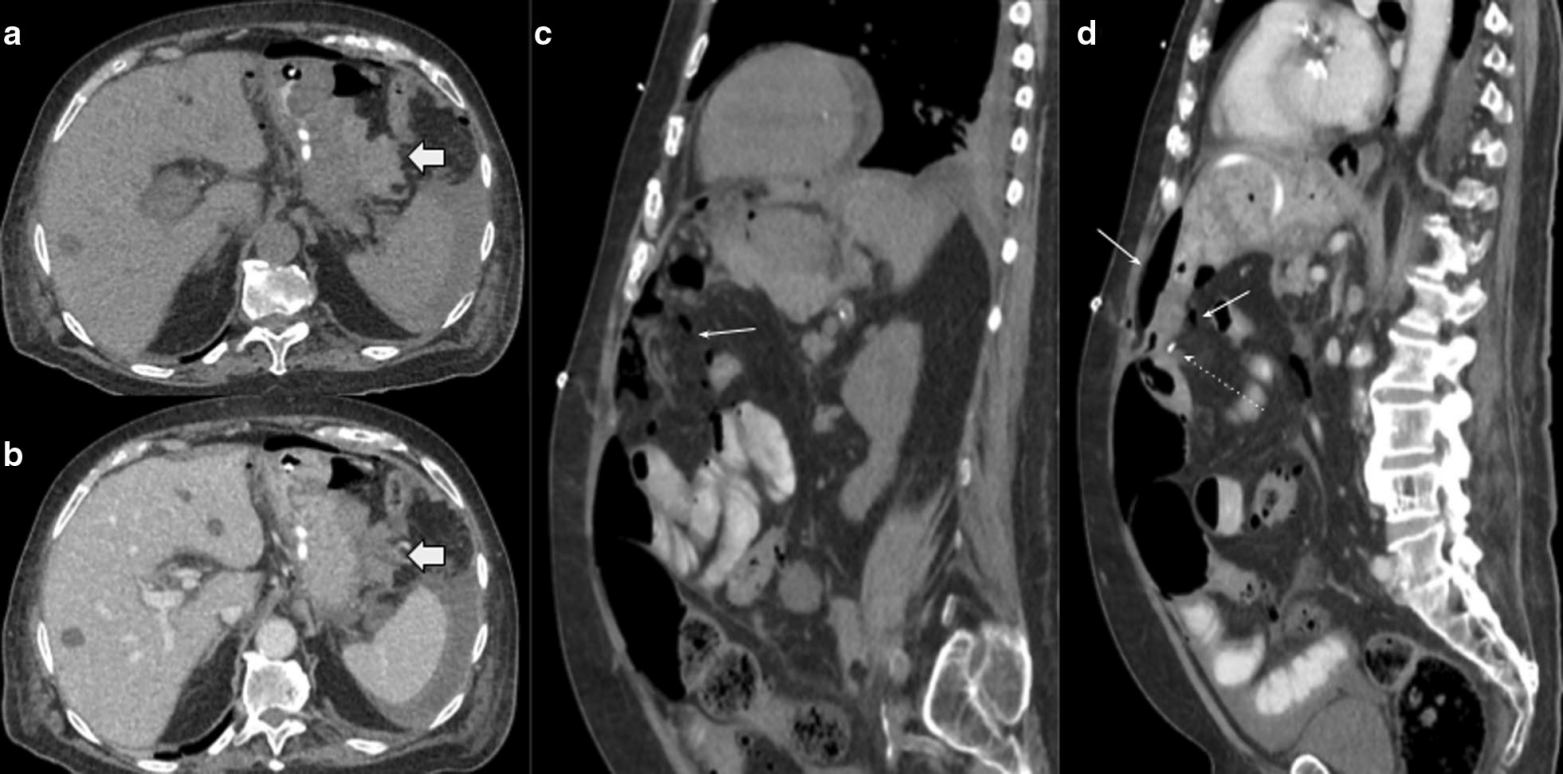
Gastrojejunostomy dehiscence. 87-year-old male patient with complicated early postoperative duodenopancreatectomy with Y de Roux and gastrojejunostomy due to pancreatic adenocarcinoma. Axial and sagittal non-contrast (**a**–**c**) and portal venous phase (**b**–**d**) CT scan. Shows a blood collection (big arrows in** a** and** b**) associated with pneumoperitoneum (thin arrows in** c** and** d**) adjacent to the gastroenteric anastomosis (dotted arrow in** d**). Urgent laparoscopy confirmed gastroenteric anastomosis dehiscence

Regarding gastrojejunostomy, delayed gastric emptying (DGE) represents a major complication after pancreaticoduodenectomy. Although it is a clinically diagnosed complication, imaging may suggest the diagnosis and dismiss others (Fig. 23). The pylorus-preserving procedure was thought to reduce DGE and avoid the biliary reflux from the afferent limb, improving long-term patient nutritional status. Nevertheless, there is no strong evidence of difference between this procedure and Whipple’s [18]. Anastomotic dehiscence is a rare and life-threatening complication related to gastrojejunostomy (Fig. 24).

**Fig. 25:**
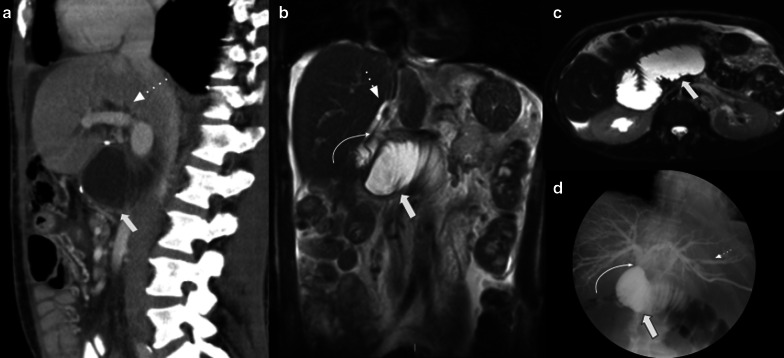
Afferent limb syndrome. 55-year-old male patient with Y de Roux reconstruction during abdominal pain and jaundice. Multimodality imaging assessment revealed marked distension of afferent limb (big arrow). Hepaticojejunostomy was permeable (curved arrow in **b** and **d**), and intrahepatic biliary dilatation is also noted (dotted arrows in **a**, **b** and **d**). Surgery confirmed afferent limb syndrome caused by limb stricture near entero-enteric anastomosis

Another clinical scenario may be present when afferent limb is partially or completely obstructed, developing afferent limb syndrome. This syndrome is characterized by mechanical obstruction of afferent limb, may be acute or chronic depending on grade of obstruction. Abdominal pain, nausea, vomiting, bacterial overgrowth, diarrhea, malabsorption and undernutrition are its usual clinical presentation resulting in pancreatobiliary disturb (Fig. 25). It is a frequent complication after Y de Roux reconstruction, described at least in 13% cases [19].

#### Vascular complications

**Fig. 26:**
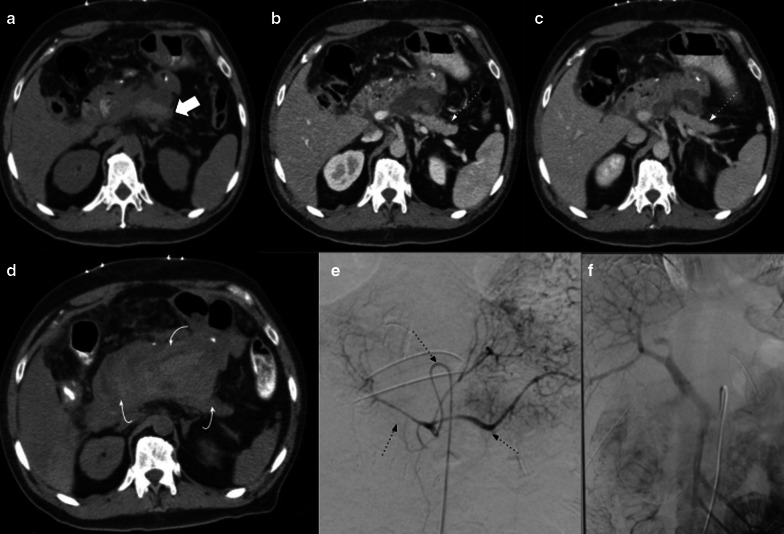
Postoperative hemorrhage. 67-year-old patient during early complicated postoperative.** a**–**c** Axial non-contrast, arterial and portal venous phase, respectively. Shows blood collection in the surgical bed (thick white arrow in (**a**), no active bleeding was seen. Remnant pancreas was preserved (dotted arrow in **b** and **c**). (**d**) CT scan performed three days later due to decreasing hematocrit shows known hematic collection increased in size (curved arrows). (**e**, **f**) Digital angiography shows no signs of active bleeding, but there is diffuse spasm of the celiac trunk (dotted black arrows in** e**) Portal-mesenteric veins were preserved. Exploratory laparotomy confirms hemoperitoneum with retroperitoneal branch-dependent bleeding

Hemorrhagic complications are infrequent (2–15%), with a high mortality rate [20]. They usually develop during the early period after surgery (< 1 week). The inadequate ligation of gastroduodenal artery during surgery is a leading cause of active bleeding. Multiphase CT scan is an excellent method to depict vascular complications: the presence of dense collection on non-contrast CT suggests hematic collection, whereas a dense area seen on arterial phase, which increases in late ones, is consistent with active bleeding [21] (Fig. 26).

**Fig. 27:**
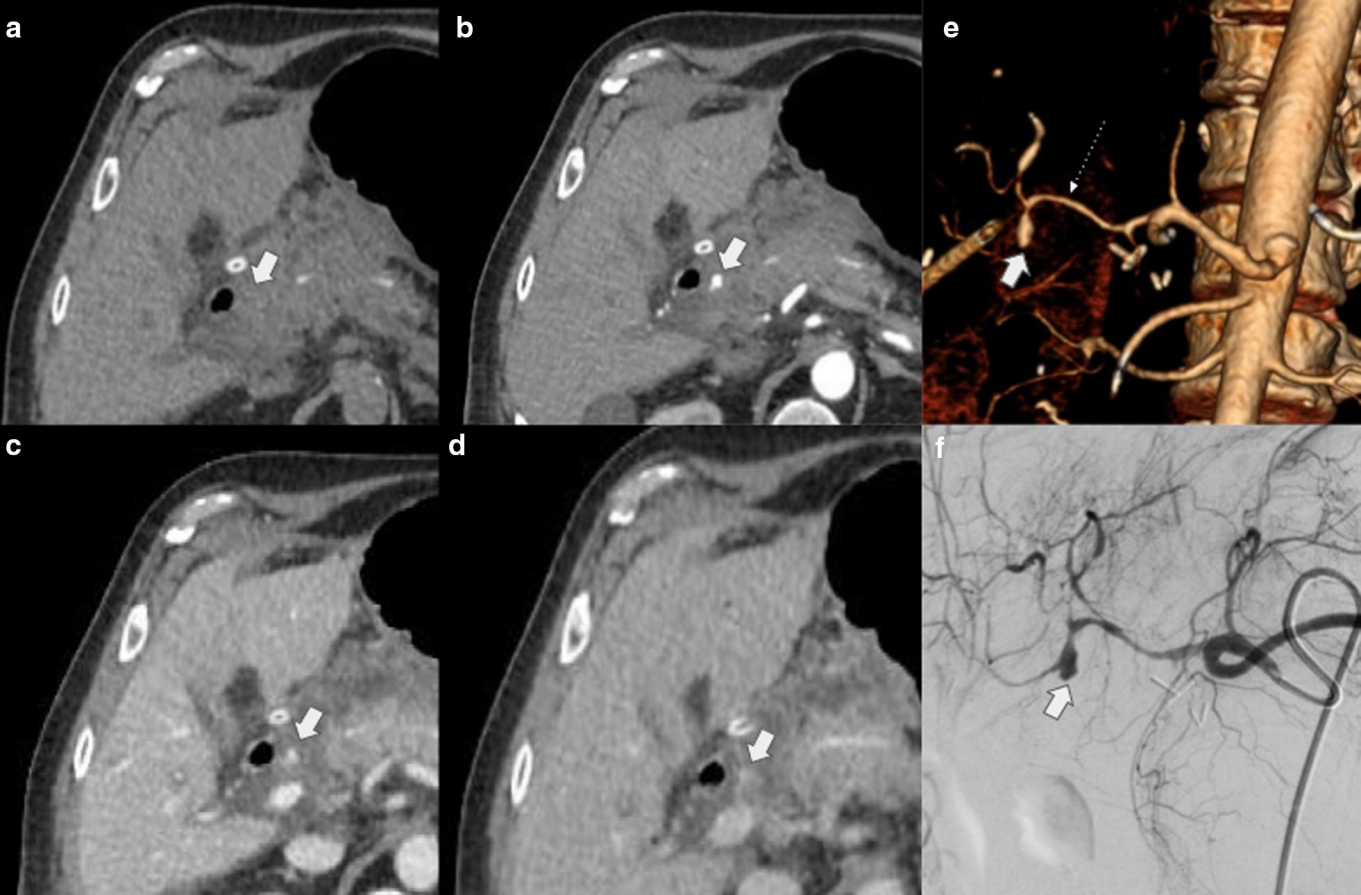
Right hepatic pseudoaneurysm. 78-year-old male patient with a history of pancreaticoduodenectomy with right hepatic artery reconstruction for pancreatic adenocarcinoma under study due to hematocrit decrease. (**a**, **b**, **c**, **d**) Multiphase CT shows a focus of focal enhancement in arterial phase in the surgical bed (arrow in **b**), which persists in post-arterial phases (big arrows in **c** and **d**). (**e**) 3D reconstruction shows saccular dilatation (arrow) dependence of the right branch of the hepatic artery (dotted arrow). (**f**) Digital angiography confirms the diagnosis of pseudoaneurysm

Direct vascular injury either from iatrogenic trauma or vascular erosion due to anastomosis dehiscence may develop pseudoaneurysm, which represents a challenging diagnosis with a high potential mortality rate if untreated (Fig. 27).

**Fig. 28:**
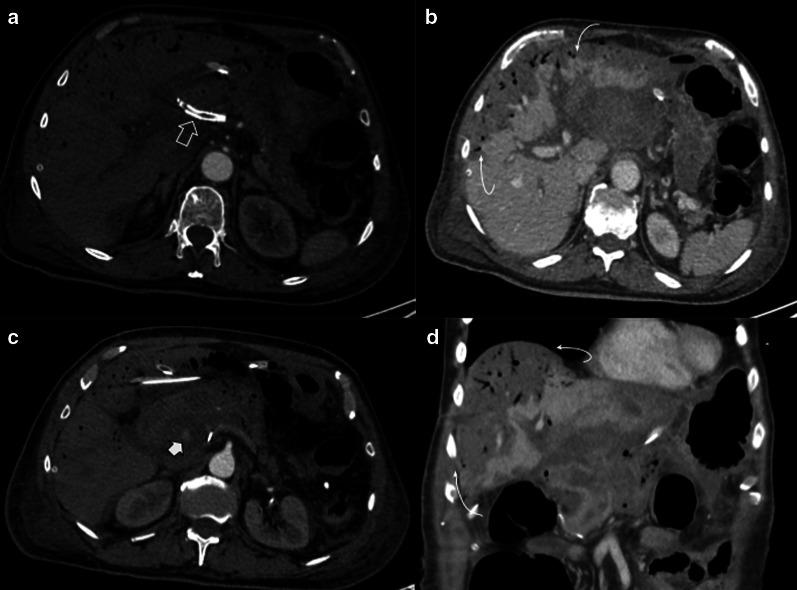
Hepatic infarct. 78-year-old patient with complicated early postoperative due to pseudoaneurysm. Stent in the hepatic artery was placed. Control CT scan due to increased hematic fluid drainage evidence occluded stent (blank arrow in **a**) and big subcapsular hypodense areas in liver parenchyma (curved arrows in **b** and **d**) consisting with infarct. An active bleeding focus depending on the ligated gastroduodenal branch was also detected (big arrow in **c**)

Vascular injury also may determine decreasing blood flow to certain organs with infarct development (Fig. 28).

## Conclusion

The pancreaticoduodenectomy procedure is associated with several postsurgical complications, knowledge of which is essential. Radiologists should be able to depict the principal anatomy landmarks and normal post-procedure findings, as well as imaging findings of each possible complication in order to achieve the correct diagnosis.

## Data Availability

Not applicable.

## Abbreviations

BF: Biliary fistula
DGE: Delayed gastric emptying
DJ: Duodenojejunostomy
DWI: Diffusion-weighted imaging
GJ: Gastrojejunostomy
MDCT: Multi-detector computed tomography
MRCP: Magnetic resonance cholangiopancreatography
MRI: Magnetic resonance imaging
PF: Pancreatic fistula
PG: Pancreaticogastrostomy
PJ: Pancreaticojejunostomy
